# Treatment of sepsis: a systematic review of its main concepts

**DOI:** 10.1186/cc12962

**Published:** 2013-11-05

**Authors:** Jéssica Santana, Magna Angélica Alves, Edwin Rodrigo Paiva Borges, Beatriz Murata Murakami

**Affiliations:** 1 Faculty of Nursing, Hospital Israelita Albert Einstein, Sao Paulo, Brazil

## Background

Sepsis is a major challenge in medicine, its high incidence, mortality and high costs making this syndrome the leading cause of mortality in ICUs, and is considered a health problem in a worldwide extension that affects millions of people and results in high morbidity and mortality. It is believed there are 18 million annually reported cases, and of every four people diagnosed one is victimized by sepsis [1-3]. The aim of this study was to identify the main aspects in the treatment of sepsis in the last 10 years.

## Materials and methods

A quantitative, descriptive and cross-sectional, literature study, concerning the main aspects in the treatment of sepsis. A semi-structured instrument developed by the authors was used to collect data to categorize the studies obtained. After collection, an electronic spreadsheet was generated, and data were analyzed using descriptive statistics.

## Results

Ten studies with a central theme focused on the treatment of sepsis were used. Seventy percent of these studies were between the years 2008 and 2011. Fifty percent of the articles mentioned that the early approach of the infectious agent is very important for successful treatment, while 60% reported that the control of the infectious focus is one of the main alternatives. Fifty percent of the studies also reported an infusion of antibiotics in accordance with the infectious focus as essential to the treatment of sepsis, and 80% reported the use of activated protein C as an indicator for diagnosis septic patients. It is observed that most studies seek early detection of the infection and early antibiotic administration, which reinforces the need for optimization of processes for the bundle of the first hour of the sepsis protocol proposed by the Latin American Institute of Sepsis (ILAS).

## Conclusions

The present study therefore concludes that for effective treatment of sepsis an early approach right after diagnosis of the disease is indispensable. Likewise, the treatment of sepsis primarily seeks to control the infectious focus using specific antibiotics. Also, the use of activated protein C may be a good alternative in the diagnosis of this pathology and a good indicator for controlling this disease.
